# Dr. Fred Rice

**Published:** 1918-08

**Authors:** 


**Dr. Fred Rice** of Ripley was fatally injured on June 21st when his auto was struck by a Buffalo & Lake Erie traction car near Westfield. He graduated from the Buffalo University in 1902.

